# Recycling of Plastics from Cable Waste from Automotive Industry in Poland as an Approach to the Circular Economy

**DOI:** 10.3390/polym13213845

**Published:** 2021-11-07

**Authors:** Dorota Czarnecka-Komorowska, Wiktoria Kanciak, Mateusz Barczewski, Roman Barczewski, Roman Regulski, Dariusz Sędziak, Cezary Jędryczka

**Affiliations:** 1 Faculty of Mechanical Engineering, Poznan University of Technology, 60-965 Poznan, Poland; wiktoria.kanciak@student.put.poznan.pl (W.K.); mateusz.barczewski@put.poznan.pl (M.B.); 2 Institute of Applied Mechanics, Poznan University of Technology, 60-965 Poznan, Poland; roman.barczewski@put.poznan.pl; 3 Institute of Mechanical Technology, Poznan University of Technology, 60-965 Poznan, Poland; roman.regulski@put.poznan.pl (R.R.); dariusz.sedziak@put.poznan.pl (D.S.); 4 Institute of Electrical Engineering and Electronics, Poznan University of Technology, 60-965 Poznan, Poland; cezary.jedryczka@put.poznan.pl

**Keywords:** polymer cables waste, recycling, compatibilization, mechanical properties, surface morphology, water absorption, circular economy

## Abstract

This paper presents the contemporary problems of polymer waste recycling, mainly recycling cables from end-of-life vehicles. The authors developed a new material based on mixed polymer waste (ASR) modified with a ductile polymer, mainly recycled low-density polyethylene (rLDPE), to produce moisture-resistant boards with beneficial mechanical properties. The ASR-based compositions without and with homogenization process—including physical, chemical, and shear-assisted compatibilization—were successfully applied and verified by evaluating final recycled product properties. The results showed that recycled polyethylene (rLDPE) was effective as a modifier increasing tensile modulus and flexural strength compare to an ASR mixture. It was found that the adding 5 wt % of polyethylene-grafted maleic anhydride (PE-g-MAH) as a compatibilizer to the ASR mixture significantly increases the homogenization of the components in the ASR matrix. The optimal solution for management cable waste is the manufacture of ASR composites with homogenization using an internal mixer the adding 20 wt % of rLDPE and 5 wt % of PE-g-MAH to the mixed plastics cable waste. The results obtained demonstrate that the hot-pressing with the pre-blending with rLDPE and compatibilizer of the ASR based waste provides a high gain in mechanical and usage properties, enabling the circular economy of plastics from automotive cables.

## 1. Introduction

Currently, plastic recycling is necessary to save the world from the increasing degradation of the natural environment [1,2] and to reduce the production costs of plastic products [3,4,5]. Moreover, recycling is an important method of polymer waste management, which allows for the recovery of secondary raw materials in the form of regranulates or chemical raw materials, which, according to the circular economy (CE) principle, can be re-included in a closed material cycle [6,7]. One definition says that CE is an industrial system focused on closing the loop for material and energy flows [4]. An alternative CE could be closed loops in industrial ecosystems by applying a reduce–reuse–recycle principle that prevents the generation of plastics wastes and turns wastes into resources [8,9]. High value-added recycling is the next key step to a circular society [10]. Zhang et al. [10] show that whether or not waste is recycled relates to multiple factors, such as end-of-life conditions, the function of materials, marketing of secondary materials, and efficiency of a treatment process [10].

However, waste management encounters numerous difficulties, firstly, recycling entails an unfavorable change in the strength and processing properties of recyclates [11,12]; secondly, crosslinked polymers are not suitable for reprocessing [13,14].

Problems arise with the recycling of the different polymers mixed. One of the most commonly used methods for higher end mechanical and/or thermomechanical performance features of immiscible polymeric blends is physical modification [15,16,17,18], consisting of the introduction of virgin materials, fillers, or the addition of ductile polymers. The second problem can be solved by introducing crosslinked polymers as fillers in thermoplastic polymer matrices or using coupling agents and compatibilizers to improve the miscibility of components [19]. Compatibility is, therefore, a process of stabilizing the phase structure of a system of thermodynamically incompatible polymers, usually due to incorporating an additional component (compatibilizer) that binds separate phases [20]. The most frequently used compatibilizers are polymers functionalized by grafting unsaturated functional monomers—i.e., maleic anhydride or acrylic acid [21]. Studies published studies thus far have showed that applying a suitable compatibilization method might be successfully used to modify the reclaimed rubber mixture containing various thermoplastics [22,23]. Furthermore, the addition of dedicated coupling agents improves the miscibility and interfacial adhesion of ingredients, thus improving the mechanical performance of final waste material-based products [23].

Among the effective compatibility methods of compositions containing rubber wastes, mixing them with appropriately selected thermoplastic polymers, which can be referred to as physical compatibilization, should also be mentioned [24]. The solutions described in the literature—such as the use of waste polyethylene [25,26] and the incorporation of elastomers such as styrene-butadiene-styrene (SBS) or block copolymers [27] into multi-polymer compositions—showed high efficiency, and therefore can be defined as justified from an industrial point of view.

Kollár, M. [28] proved that, a blend of poly-vinyl-chloride (PVC) with polyethylene (PE) could be prepared by melt blending using low- and high-density polyethylene without applying a compatibilizer. Stable microheterogeneous materials were obtained, despite the PVC and the PE being incompatible polymers. The decrease of glass-transition temperature and energy of activation showed that there was a slight mixing of the polymers. It has been found that a small amount of PE dissolves at the molecular level and can act as a plasticizer in PVC, greatly increasing molecular mobility [28].

Also Marossy [29] proved that the low polyethylene content in the PVC mixture improves the stiffness. According to Arnold [30], impurities in the form of PE accelerate the process of PVC degradation due to the presence of free radicals formed during thermo-oxidative degradation of PE. For example, Fang [31] cross-linked PE in the melt state to avoid phase separation of polyethylene and polyvinyl chloride.

The compatibility of nonpolar PVC polymers can be improved by grafting; for example, Hou and Zhao [32] have successfully mixed PVC with PP using grafting technique or by adding various compatibilizers—i.e., chlorinated polyethylene (CPE), nitrile rubber (NBR), and polyurethane [33].

Most of the conducted research focuses on developing better technological and organizational solutions to recover as many material fractions as possible. In addition, numerous scientific studies are conducted on developing a method to improve the properties of polymer recyclates and find appropriate industrial applications for them. In Poland, the level of waste recovery in 2020 was only 25%, which is still very little compared to the leaders in Europe. Germany and Scandinavia recover over 80% of polymeric waste. A significant group of waste generated in Poland is waste from end-of-life vehicles under the ELV Directive, including electric cables, car adapter cables, and cable plugs of which about 30% are plastic components [34,35].

One of the most critical stages in waste management from the perspective of costs as recovery efficiency is segregation and separation of waste [36,37]. In automotive shredder residue (ASR)—including cables, electrical harnesses, and plugs—the problem is even more remarkable because there is a wide variety of plastics, from classic pure thermoplastic polymers to crosslinked plastics, numerous mixtures, and composites with fillers, i.e., glass and carbon fibers, talc, calcium carbonate, and basalt derivatives. In order to reduce the costs related to segregation, it can be proposed to eliminate expensive separation in favor of processing mixed wastes into full-value products with the use of the extrusion, rotational molding, or hot-pressing technology. Xanthos et al. [38] proposed mixing ASR with low-density polyethylene (LDPE) and forming in one-step processing blocks by intrusion process. While used by them composition contains mostly highly crosslinked rubber with high calcium carbonate content; the ground ASR was instead treated as rigid filler of LDPE-based composites that polymeric blend. Diaz and Ortega [39] presented an interesting concept with co-workers who used rotational molding to reprocess polymeric cable waste. It is known that simultaneous physical and chemical modification causes the multiplication of plastics’ mixing effect, thus obtaining homogeneous properties of composite products [27]. This opens up new opportunities for the efficient material recycling of mixed polymer waste from the automotive industry. In most cases, the reprocessing of waste materials is accomplished through melt processing with extrusion, injection, and compression [40]. However, it should be emphasized that it is essential to reduce the number of times the processing of waste materials is possible so that the total energy consumption during reprocessing is as low as possible.

In the case of mixtures containing ASR cable waste, a physical modification by adding a recycled polyethene low density (rLDPE) and polyethylene-grafted maleic anhydride (PE-g-MAH) as a compatibilizer was carried out. ASR composites were subjected to mixing in an internal mixer, providing the appropriate shear stresses needed to distribute and dispersion the components of the composition. In this paper, the authors developed a new material based on mixed polymer waste modified with an rLDPE, to produce moisture-resistant boards and wall protective cladding for animal safety with beneficial mechanical and usage features.

The work aimed to develop a new solution in recycling mixed cable polymer waste by developing a suitable processing and modification procedure.

## 2. Materials and Methods

### 2.1. Material Preprocessing and Identification

**Figure 1:**
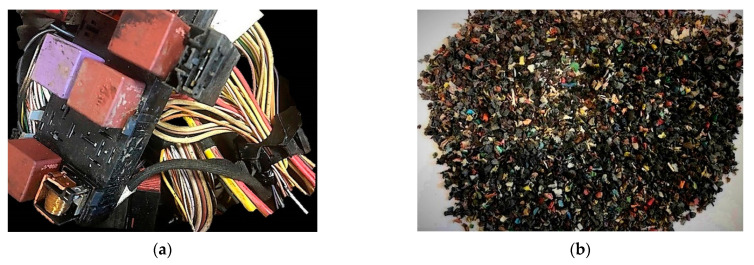
Origin wastes: (**a**) automotive originated cables wastes; (**b**) recovered and separated polymeric wastes of 1.8 mm fraction.

The research subjects are ASR plastic waste obtained from used cables and car bundles according to the technology developed by Euro Metall (Katowice, Poland). Figure 1 shows the electric cable bundles (Figure 1a) subjected to the preprocessing, including grinding and separation processes, resulting in a pure polymer fraction (Figure 1b), devoid of waste such as copper and aluminum. Wastes of cables contain several different polymers—including mainly polyvinylchloride (PVC), natural rubber, polyurethane (PU), polyethylene terephthalate (PBT), polyethylene (PE), hydrogenated styrene butadiene rubber (HSBR), and polypropylene (PP). The content of mentioned polymeric grades was confirmed by the realization of Fourier transform infrared (FTIR) spectroscopy using a Jasco FT/IR 4600 spectrometer (Jasco Europe S.R.L., Cremella, Italy)), the results of which are presented in Supplementary Data in Figures S1–S13. Additionally, Table S1 presents the results of the curve fitting software developed using the Spectra Manager TM software (ver. 2, Jasco, Easton, MD, US), along with the assigned degree of the fitting from 30 measurements for each series. The mentioned polymers are typically used to manufacture cable insulation and electrical connectors. The noticeable presence of rLDPE was noted only in M4−M6 compositions containing intentionally introduced polyethylene as a physical compatibilizer.

PE-g-MA is a compatibilizer for polymer blends which serves as support for polar to nonpolar substances [41]. The compatibilizing agent PE-g-MAH (viscosity 1.700–4.500 cP at 140 °C, T_m_ (DSC) = 105 °C, density 0.9 g/cm^3^ at 25 °C) [42] was purchased from Sigma–Aldrich (St. Louis, MO, USA). Polyethylene (rLDPE) from post-consumer waste was obtained from a local Polish supplier.

During preliminary investigations, the ground ASR polymer wastes were characterized with a quantitative particle size analysis Vibratory Sieve Shaker Fritsch ANALYSETTE 3 Pro apparatus (FRITSCH GmbH-Milling and Sizing, Idar-Oberstein, Germany) operated in the range of 50–2800 µm, operating with AUTOSIVE software version 1.7 (Fritsch GmbH-Milling and Sizing, Idar-Oberstein, Germany).

**Figure 2:**
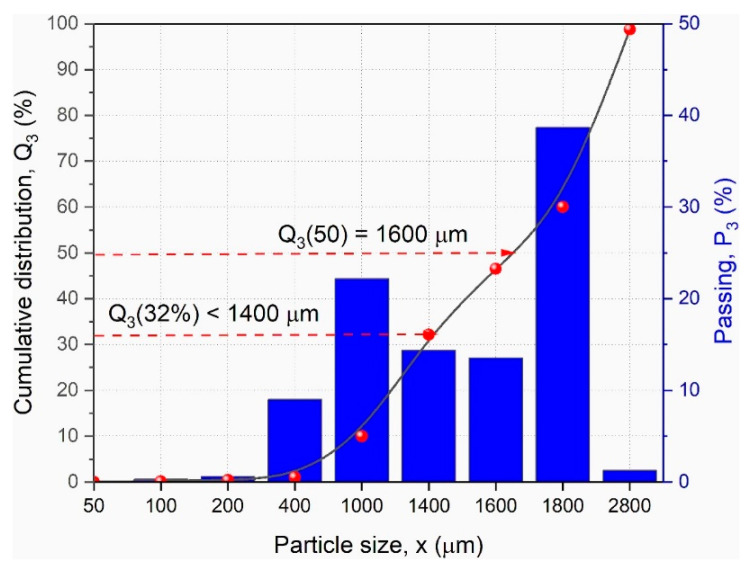
Cumulative distribution curve of the fractions (Q3) and percentages of the fractions (P3) of the ASR mixture used in this study.

The fraction of mixed polymers prepared in this way was subjected to initial identification (Figure 2); additionally, the size of the fraction and the sum of the calculated screenings were determined. 

The fractions’ mass percentages (P3) can be graphically shown as histograms (Figure 2, column graph). In this ASR mixture used, the largest fractions can be found between 1600 μm and 2800 μm with 39%.

The cumulative distribution curve (Q3) was achieved (Figure 2, line graph) by adding up the single fractions and through interpolation between the measuring points. Taking a look, for instance, at the particle size of 1400 μm the corresponding value of 32% can be read off the *y*-axis which means that 32% of the total sample is smaller than 1400 μm, as shown in Figure 2. Furthermore, the corresponding particle size (1400 μm) is read off the *x*-axis to find out the median Q3 (50) of this distribution. This means that 50% of the sample mass is equal to or smaller than 1600 μm.

### 2.2. Melt Processing and Sample Preparation

**Figure 3:**
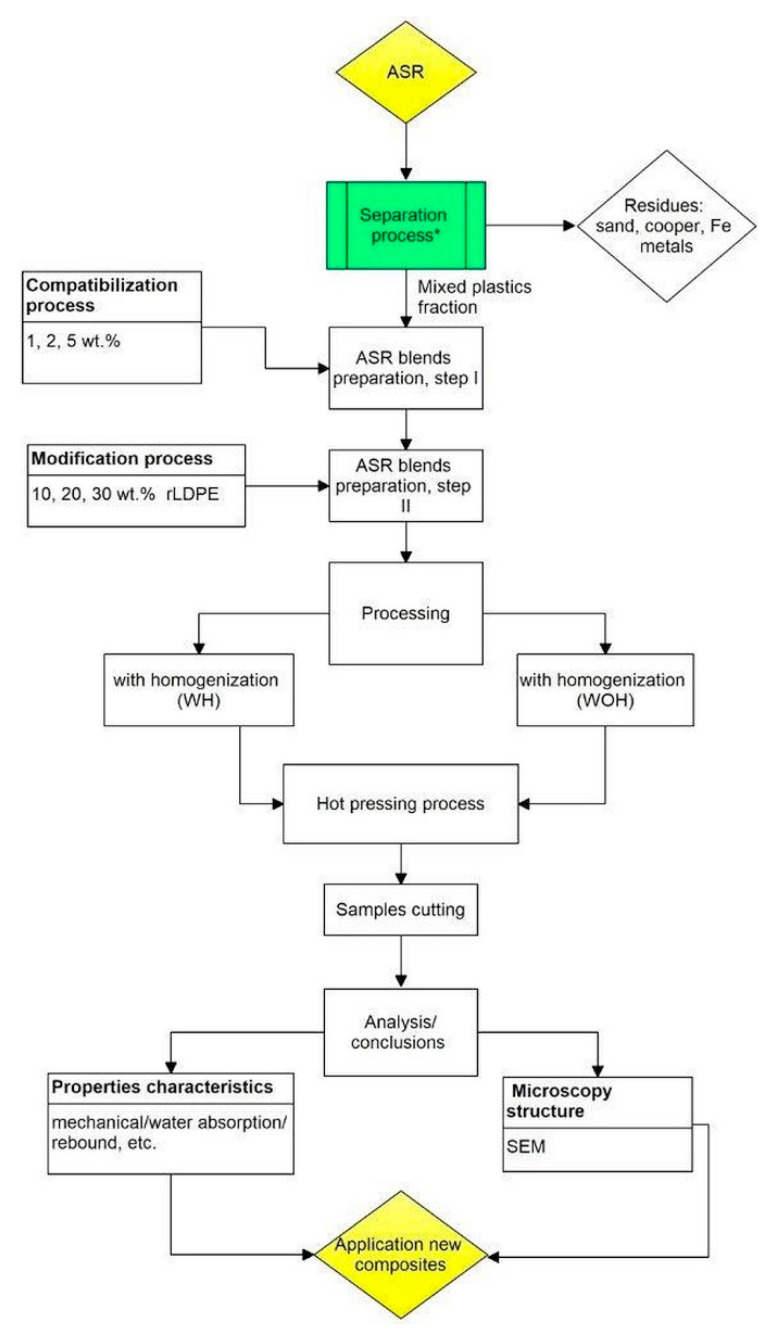
Scheme of the experimental procedure for ASR polymer composite. * the process developed by Euro Metall (Piotrowice, Poland).

In order to produce final products based on ASR mixed waste for industrial applications, six different material compositions were prepared in two series, such as without homogenization (abbreviation WOH) and with homogenization in high-shearing conditions using a mixing chamber with the delta-shaped blades (abbreviation WH). The scheme of the experimental procedure is shown in Figure 3.

In addition, due to the differences in the melting point of individual components, a compatibilizer (PE-g-MAH) based on recycled polyethylene (rLDPE) was added to the ASR mixtures to increase the degree of dispersion and adhesion between fractions of different polymer grades. As a result, ASR mixtures with the compositions shown in Table 1 were obtained.

**Figure 4:**
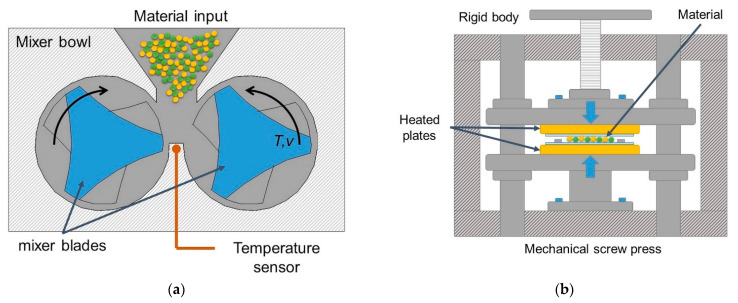
Schemes of ASR polymer composite preparation: (**a**) internal mixer; (**b**) hot pressing process.

All samples assigned as WH were melt-mixed using an internal mixer (Brabender Measuring Mixer) (Brabender GmbH & Co. KG, Duisburg, Germany), characterized with a 50 cm^3^ electrically heated chamber [43], as presented in Figure 4a. The melt processing was conducted at 190 °C, with 30 rpm for 8 min. A mixer including of two intermeshing rotors type mixer blades is situated in a controlled chamber. The mixing temperature was selected on the melting point of polyethylene (T_M_ = 125 °C). After being removed from the mixing chamber, the ASR-based compositions were pressed into 4 mm nominal thickness sheets and 100 × 100 mm^2^ nominal dimensions using a PLHS-7 laboratory hydraulic hot press (Figure 4b). Then, standardized samples were cut from these sheets by means of punching dies. The compression molding parameters are following: the melting temperature of 190 °C, melting time of 8 min, forming time of 6 min, pressure of 10 MPa, cold pressing of 10 min, and cooling time of 20 min.

### 2.3. Characterization of ASR-Based Compositions

#### 2.3.1. Density and Water Absorption

The density was determined using the hydrostatic method (according to the standard ISO 1183-1: 2013 (AXIS AD50-AD200, AXIS, Gdańsk, Poland). Ethyl alcohol (as an immersion liquid) was used, and measurements were made of five samples from each series. The water absorption tests were carried out as per PN-EN ISO 62:2008 test method. All samples were oven-dried before its weight was noted as the initial weight of the samples. The measurements were performed in distilled water, for 60 ± 2 mm square samples at temperature of 25 °C for 24 h. After the immersion time, the samples were removed from the water, dried, and weighed to the nearest 0.01 g. The amount of water absorbed by the ASR composites (WA) was calculated according to Equation (1)

(1) $$\mathrm{WA}\;=\frac{m_{1}-{\;m}_{0}}{m_{0}}\times 100\%$$

where: WA is the water absorption mass fraction (%), m_0_ is the mass of the test specimen after drying (g), m_1_ is the mass of the test specimen after immersion (g).

#### 2.3.2. Tensile and Hardness Testing

The mechanical properties of the blends were determined by tensile tests performed using a Zwick/Roell Z010 universal testing machine (Zwick GmbH & Co. KG, Ulm, Germany) and analyzed by testXpert II software. The specimens for mechanical testing were dumbbell-shaped, according to the PN-EN ISO 527-2:2012 standard. Tensile characteristics were measured at room temperature with a crosshead speed of 50 mm/min. Young’s modulus and tensile strength were evaluated from the tensile stress–strain curves. The reported data were the average of the results of five specimens. Flexural strength was measured using a three-point bending test according to PN-EN ISO 178 with a 160 mm span and a load speed of 10 mm/min (Zwick/Roell Z010 (Ulm, Germany), and then the measurements were calculated (testXpert II, Zwick, Ulm, Germany).

The hardness of samples was measured using a Shore hardness tester (HBD 100–0, Sauter GmbH, Balingen, Germany) according to the PN-EN ISO 868:2004. The hardness was indicative of an average penetration value (Shore degrees on the D scale) based on five readings from tests.

#### 2.3.3. SEM Morphology Testing

The fractured surfaces of the ASR polymer composite grafted by PE-g-MAH without and with homogenization was investigated with a MIRA3 scanning electron microscope (TESCAN Brno, s.r.o., Brno, Czech Republic) with high-resolution imaging. The specimens were fractured in liquid nitrogen and then coated with a layer of 40 nm carbon powder. The internal structure changes after homogenization process of rLDPE in the ASR matrix were investigated by back-scattered electrons (BSE) signal, with an accelerating voltage of 20 kV.

#### 2.3.4. Thermomechanical Analysis

Dynamic Mechanical Thermal Analysis (DMTA) using a MCR 301 apparatus (Anton Paar GmbH, Graz, Austria) operating in the torsion mode. The samples were heated from 25 °C to 120 °C with a rate of 2 °C/min. The strain of 0.01% was applied at a frequency of 1 Hz.

#### 2.3.5. Rebound Resilience

The rebound resilience of ASR samples was determined with a Schob type pendulum Zwick 5109 (Zwick GmbH & Co. KG, Ulm, Germany) in accordance with the ISO 4662:1986 standard. Each evaluation was prepared for 15 specimens.

#### 2.3.6. Acoustic Properties

The sound absorption coefficients of the waste-based compression-molded materials were determined using the standing wave method according to EN ISO 10534-1:1996 standard. This method does not require testing in special chambers. The use of a standing wave apparatus enables the determination of the sound absorption coefficient under well-defined and controlled measurement conditions [44]. The sound absorption coefficients were determined in octave bands: 125, 250, 500, 1000, 2000, and 4000 Hz. Two measuring tubes were applied in the test. A tube with a diameter of 100 mm was used for testing in the octave bands from 100 Hz to 1 kHz, while a tube with a diameter of 30 mm in the octave bands 2 Hz and 4 kHz. Considering the measurement methodology as mentioned earlier, the samples for acoustic tests were cut into cylindrical shapes with diameters of 100 mm and 30 mm and thickness of 8 mm. Detailed information about the signal processing procedure has been described in our previous work [45].

## 3. Results and Discussions

### 3.1. Density and Water Absorption Analysis

**Figure 5:**
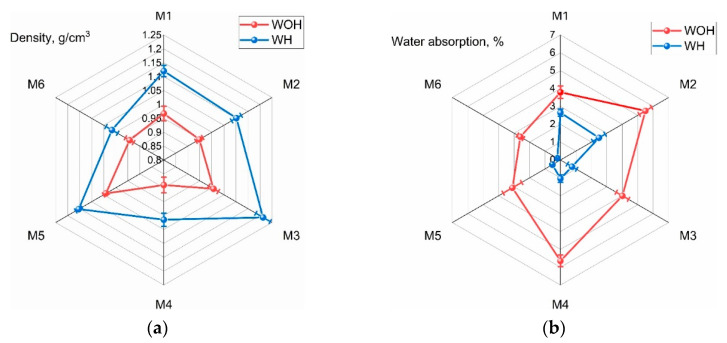
Density (**a**) and the water absorption (**b**) of ASR-based blends test results in distilled water at 25 °C.

The measured density values of final ASR-based materials prepared with a various routes of homogenization are presented in Figure 5a. Generally, we can see that the density of ASR composites increases with the addition of PE-g-MAH compared to ASR. The density tests showed that the homogenization process significantly increases the density of the obtained composites, which results from the better dispersion of the composition components in the mixing process using two intermeshing rotors. Mixing in this type of mixer is due to shear stresses in the mass of polymer between the lugs of the rotors and the chamber walls and, to a lesser extent, between the rotors. As for mixing proceeds, the charge gradually softens and deforms, thereby achieving higher compressibility and packing density of ASR-mixed polymers with rotors and chamber walls.

The increase in the content of the compatibilizer in the M1–M3 samples caused a higher density of final samples, which may be directly related to the increased proportion of recycled polyethylene supporting PE-gMAH in the ASR matrix. An interesting tendency can be observed in the case of compositions subjected to physical modification (M4–M6). In contrast with the grafted PE-g-MAH incorporated in the M1–M3, waste rLDPE, which is the source of physical comparison (M4–M6), has a lower MFI. Therefore, introducing 10 wt % of the rLDPE resulted in a smaller increase in density (than the introduction of 5 wt % of grafted PE). The lack of a linear increase in density in samples M4–M6 characterized by a rising rLDPE concentration, can be interpreted as follows. The content of 10 wt % was insufficient to form a continuous phase and penetrate the entire sample volume, leading to a more favorable distribution of the ASR. The content of 20 wt % rLDPE allowed lowering the viscosity of the composition under process conditions (both in the case of WH and WOH), which resulted in a product with reduced porosity. On the other hand, the reduction in density in the case of the M6 series is related not to the deterioration of the homogeneity of the composition and the increased content of pores, but to a large proportion of the polyethylene matrix with a density below 1 g/cm^3^.

The changes of water absorption for ASR blends without (WHO) and with homogenization (WH) are shown in Figure 5b.

It can be seen from Figure 5b that water absorption of ASR-based blends grafted PE-g-MAH significantly decreased with the increasing addition of rLDPE. As it is clearly seen from the testing, that the ASR composites produced by using the homogenization process exhibit a better trend compared to those without homogenized as shown above. This is due to the homogeneity of the structure under the influence of the compatibilizing agent, which reduces the interactions of the strongly bound water molecules in the polyamide particles with polar amide groups. Hence, the lower the water absorption of the ASR composite [37], the higher the dimensional stability of products made with this composite.

### 3.2. Mechanical Properties

**Figure 6:**
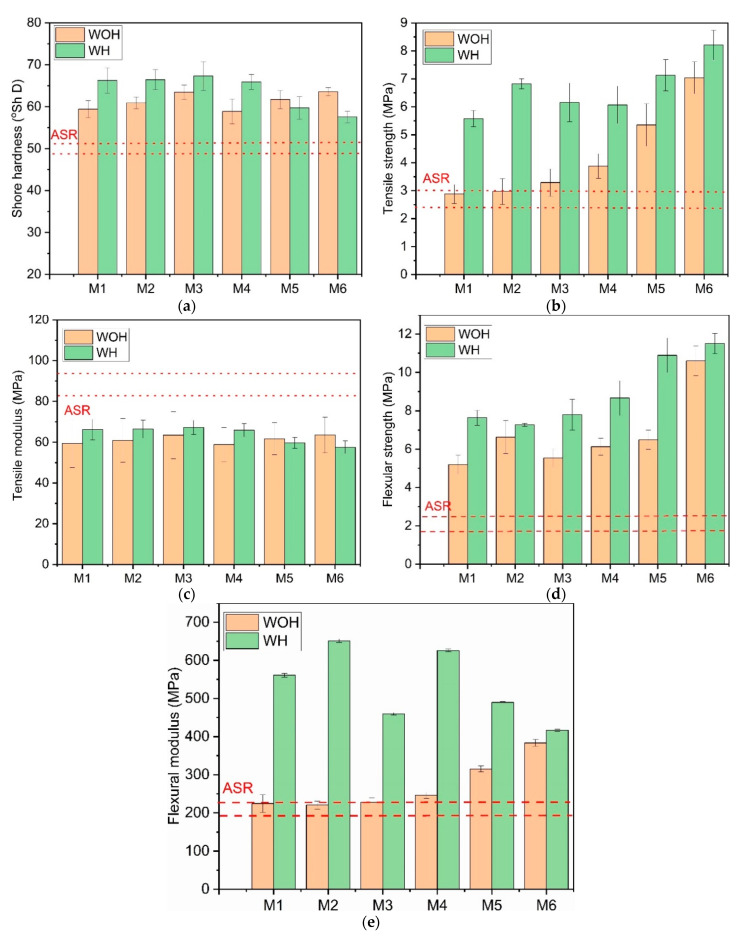
Mechanical properties of composites without homogenization and with homogenization: (**a**) Shore hardness, (**b**) tensile strength, (**c**) tensile modulus; (**d**) flexural strength, and (**e**) flexural modulus.

The effects of the homogenization process and compatibilization on the Shore hardness, Young’s modulus (E), and tensile strength of all samples are showed in Figure 6. As seen from Figure 6a, the result shows that the Shore hardness of ASR composites increased for the blends with increasing PE-g-MAH content. The increased hardness of ASR blends with the addition of a compatibilizer in relation to the components mixture may indicate the compatibilizing effect of PE-g-MAH. In the case of the ASR composites modified by rLDPE, the opposite effect was observed, which results from the presence of a ductile polymer in the ASR matrix.

Figure 6 shows the obtained results of mechanical tests for composites without (WOH) and with the homogenization (WH) process. In the static tensile test, the stress–strain curves were obtained, based on which the values of tensile strength and tensile modulus were determined.

Tensile strength for ASR blends (Figure 6b) increased when recycled LDPE was added, and increased with the addition of PE-g-MAH, indicating that a small amount of 5 wt % PE-g-MAH leads to better interfacial adhesion between the blend components [37]. As shown in Figure 6, the ASR-based compositions subjected to homogenization (WH) have significantly higher tensile (Figure 6b) and flexural strength (Figure 6d) compared to material series manufactured without preliminary mixing with high shearing conditions. For the M4, M5, and M6 series, it can be seen that higher rLDPE content increases the ability to transfer stress during static load regardless application of an additional mixing procedure. This phenomenon is understandable because increased content of low melting phase in immiscible and partially non-melting in processing conditions polymeric blend allows creating a 3D structure with ability to transfer stress during static load [25,46]. From the blends manufactured without the addition of waste rLDPE, the most beneficial tensile strength was observed for the M2 series, containing a higher compatibilizer content. As can be expected, the worst mechanical performance was noted for M1 samples manufactured without homogenization (WOH) and containing only 1 wt % of PE-g-MAH.

As seen from Figure 6b, the ASR blend modified with PE-g-MAH polyethene and a different mass fraction of rLDPE showed a comparable value of the tensile modulus. In contrast, in the case of flexural modulus (Figure 6e), the high compatibilizer amount (above 10 wt %) caused the decrease in sample stiffness. This effect may be related to the higher content of the recycled polyethylene in compositions. With this effect in mind, it is understandable that the elasticity modulus studied in both measurement geometries decreases, observed for homogenized samples with an increasing share of rLDPE.

The flexural properties of ASR-based blends prepared with various coupling methods are presented in Figure 6d–e. It can be observed that both the flexural strength (Figure 6d) and the flexural modulus (Figure 5e) of ASR composites after homogenization were significantly higher compared with those without homogenization. All material series without homogenization exhibit a higher modulus than the series modified by rLDPE processed with homogenization. It was noted that the ASR-blends with an increasing amount of rLDPE revealed lower stiffness, which agrees with the tensile modulus indication of the presence of ductile polymer content in a polymer matrix. In particular, the addition of 50 wt % rLDPE leads to lowers modulus of approx. 70%. It was also noted that the addition of rLDPE to the ASR matrix resulted in a more clear enhancement of the flexural strength compared with the compositions without recycled polyethylene content. The M6 series shows the highest flexural strength after homogenization.

### 3.3. Macroscopic Surface Morphology Analysis

**Figure 7:**
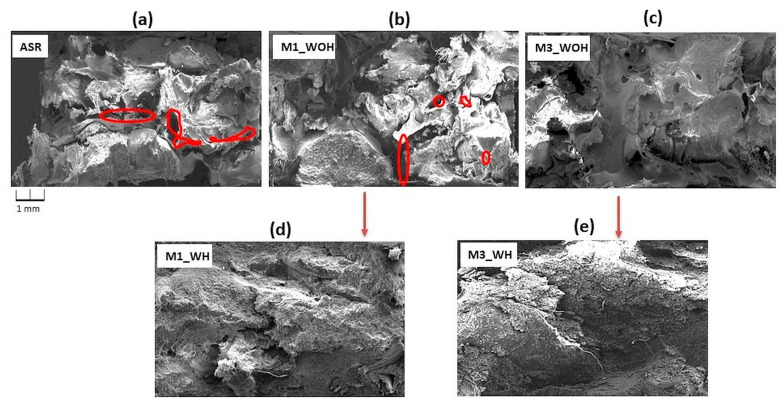
SEM images of the fractured surface ASR sample (**a**) and its composites with 1 wt % and 5 wt % compatibilizer content (**b**,**c**) without (WOH) and with homogenization (**d**,**e**) (WH).

**Figure 8:**
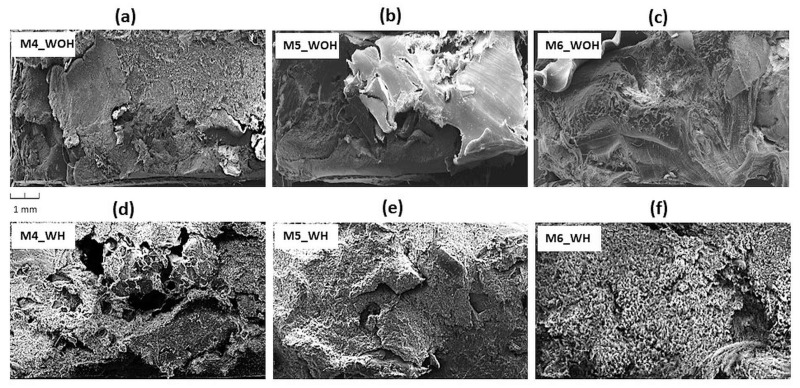
SEM images of the fractured surface ASR composites modified by different rLDPE content (**a**–**c**) without (WOH) and with (**d**–**f**) homogenization (WH).

Figure 7 shows SEM microscopic images of fractured ASR samples (a) and their composites with the 1 wt % and 5 wt % of PE-g-MAH content as the compatibilizer (b,c) without (WOH) and with (WH) homogenization process (d–e). Figure 8 shows ASR modified 10, 20 wt % and 30 wt % content of waste rLDPE (a–c), subjected to the homogenization process (d–f). As a result of the microscopic assessment of the fracture surface structure, it can be concluded that M0–M3 compositions without homogenization (WOH), Figure 7a–c are characterized by the presence of a higher amount and more distinct free spaces (red circles) in the interfacial region, which is due to enlarged interfacial tension and poor adhesion between components of ASR blend.

We can see that the ASR series (Figure 7a–c and Figure 8a–c) without homogenization (WOH) have clearly visible boundaries between the components, compared to composites after homogenization (WH), which are distinguished by a more compressed structure, free of defects and discontinuities (M5 and M6_WH). This indicates a significant improvement in the degree of dispersion under the shear forces’ influence during the mixing of components in the mixer chamber [47] and increased interfacial adhesion due to the compatibilizer [48]. Figure 8 group (M4_WH, M5_WH, and M6_WH) shows the ASR/rLDPE blends with homogenization exhibit better dispersion and distribution of rLDPE (especially in the case of M5_WH and M6_WH composites). Moreover, it was confirmed that the PE-g-MAH compatibilizer had indeed improved the compatibility between mixed plastic wastes.

### 3.4. Thermomechanical Behavior

**Figure 9:**
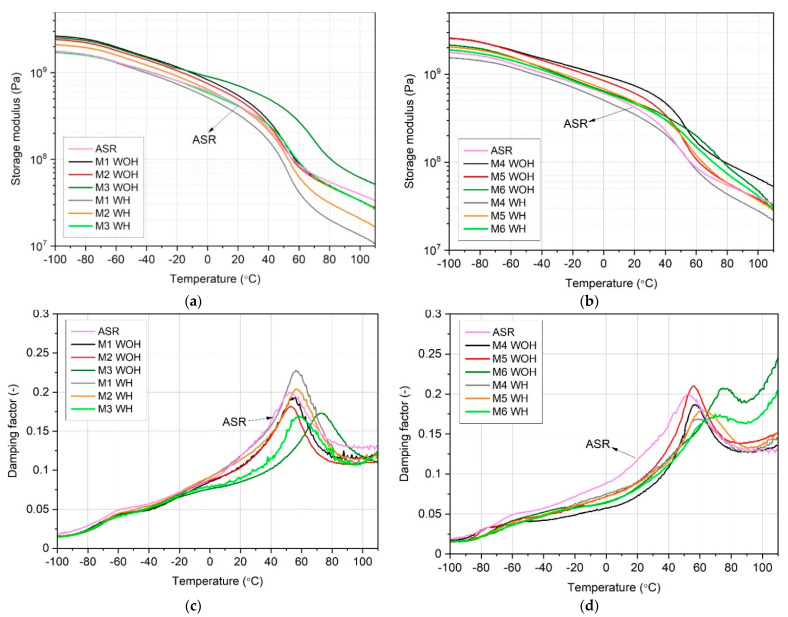
Plots of the storage modulus (**a**,**b**) and damping factor (**c**,**d**) of ASR-based compositions produced without homogenization (WHO) and with homogenization (WH).

Figure 9 summarizes the results of the dynamic thermomechanical analysis performed for ASR-based blends in the form of storage modulus (G’), Figure 9a,b and damping factor (tanδ), Figure 9c,d as a function of temperature curves. For all considered materials, there is a visible dominating peak at tanδ vs. T curves in the range between 55 °C and 80 °C originating from the PVC glass transition [49,50], and in the case of M4–M6 series also with α-relaxation of low-density polyethylene [51]. All ASR compositions containing only a compatibilizer (M1–M3) showed a similar tendency, mainly lowering the intensity of the maximum value of tanδ with increasing content of the compatibilizer. According to de Carvalho and Sirqueira [52], this phenomenon is connected with the formation of chemical bonds between mixture components, which reduce compatibilized composition damping ability at higher temperature values and confirm the compatibilization efficiency. For both M3 series similar effect of shifting the maximum of tanδ peak to higher temperature was observed. However, the sample prepared in shearing conditions showed both lower temperature at peak as well as slightly lower maximum, which suggests lower damping ability. This effect may be interpreted in a two-way manner, as a result of the lower processing temperature, which caused lower efficiency of compatibilizer or achievement of the more uniform structure of the composition. Although polyethylene itself is not characterized by high thermomechanical stability, the series containing its additives (M4–M6) were characterized by increased storage modulus values (G’) in the range of increased temperature (at 80 °C) compared to the M1–M3 series. It proves the correct course of physical compatibility, realized by introducing a highly elastic polymer into the ASR-blend. Most importantly, the results of the tests presented in Figure 8a,b are consistent because, in both cases, the highest stiffness at the maximum measurement temperature (110 °C) was shown by an M3 WOH sample with 5% content of compatibilizer, not subjected to the homogenization process. A different tendency was noted in the case of the M4 WH composition, which showed the lowest G‘ values in the entire temperature range. The increasing share of rLDPE up to 50 wt % in the structure of the W6 mixture caused a change in the nature of the curve, leading to the calm course of the drop of G‘ and shifting it from 40–60 °C to about 60–80 °C in temperature range, which is related to α-relaxation of the polyethylene.

### 3.5. Mechanical Damping and Acoustic Properties

**Figure 10:**
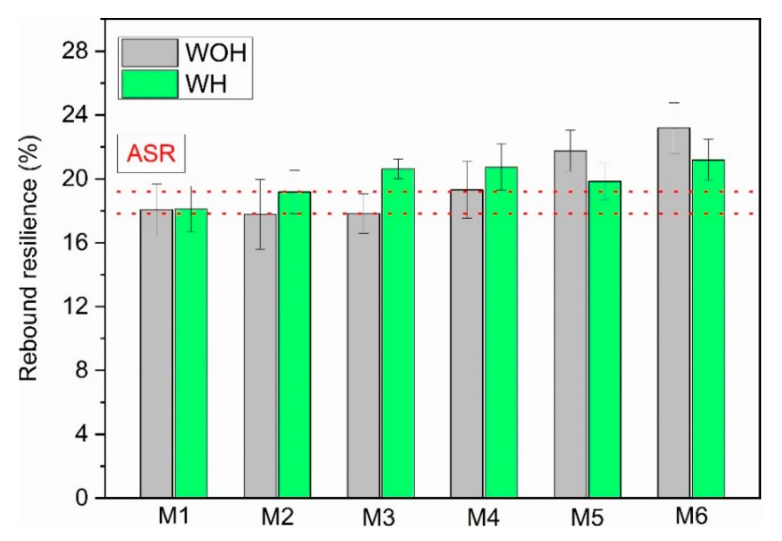
Rebound resilience (R) of ASR-based blends formed without and with homogenization.

The rebound resilience (R) changes for ASR-based compositions produced with various methods of compatibilization are presented in Figure 10. 

Non-homogenized maleic anhydride-modified samples showed comparable values for this feature, while for the WH series, materials assigned as M1, M2, and M3 showed improved R with increasing coupling content agent. When using physical compatibility (M4–M6)—i.e., introducing waste polyethylene—more intense effects of the additionally introduced polymer was noted for samples from the WH series. A partial correlation between the hardness results and the rebound resilience results can be observed in various studies [53,54]. In the case under consideration, comparing the hardness results presented in Figure 6a, the observed changes in rebound resilience are consistent with the dependence as mentioned earlier. The material series manufactured without physical compatibilization conducted by LDPE addition had higher hardness, resulting in lower R values than the physically compatibilized series. It is assumed that lower rebound resilience values are observed for samples with higher impact energy absorption ability [55]. The value of the rebound resilience determines the scope of the material’s applicability because the elements with increased stiffness and the ability to absorb impact energy may be an essential element of mechanical systems exposed to impact load. However, on the other hand, high flexibility translating into high rebound resilience values will allow for the production of materials with increased impact load resistance and the ability to absorb mechanical vibrations without the risk of damaging their structure.

**Figure 11:**
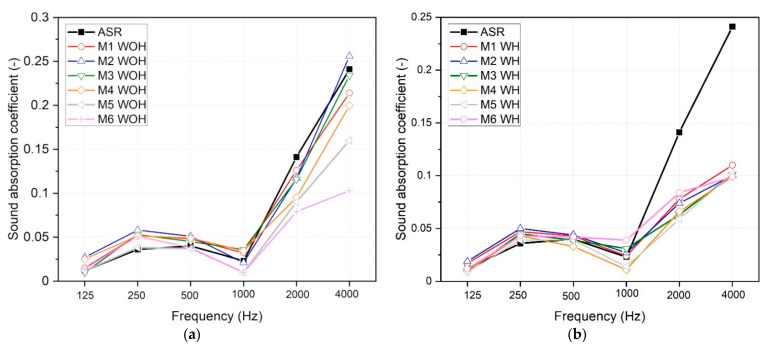
Sound absorption coefficients of ASR-based compositions prepared without (**a**) and with (**b**) homogenization.

Figure 11 presents the changes in sound absorption coefficient (α) in the frequency function for compression-molded samples based on ASR waste with and without adding PE-g-MAH compatibilizer. 

Measured values are in good agreement with data reported in the literature for samples made of rubber, PVC, or different plastomers [56,57]. The results showed, that due to the relatively low sound absorption coefficient, all the mentioned materials cannot be classified as sound-absorbing materials. While all material samples, both homogenized and those without mixing in the presence of intense shear, show relatively low and similar α values in the range up to 1 kHz, significant discrepancies between individual material series are observed in the frequency range above 1 kHz. Taking into account the results obtained for the compression-molded samples based on ASR, which is characterized by a significant heterogeneity of the structure, it can be concluded that more favorable effects in terms of acoustic attenuation are observed for materials characterized by less uniform structure (without homogenization). For all WOH compositions, a lower density than for the WH samples was also noted. Because these materials did not differ in material composition, the difference in density was due to the greater porosity of the non-homogenized materials series (Figure 4a). As shown in the different studies [58], interconnected small pores may significantly influence the sound absorption of various materials. Moreover, there is a noticeable tendency in lowering α in the high-frequency range (above 1 kHz) for the M4–M6 WOH series, along with the increasing proportion of polyethylene in the structure. This effect suggests that LDPE’s compatibilizing effect on ASR blends with its share (higher density values and filling the space between non-melting rigid particles). Colom et al. showed a similar effect in PVC-ground tire rubber (GTR) composites [57]. They discussed creating “rigid frame porous material” as an effect of microporosity development in non-compatibilized interfacial areas between PVC and GTR domains. An additional photograph of samples used for acoustic research has been compiled in supplementary information as Figure S14. Sound absorption is dependent on the thickness of the sample the thicker the sample [57,59,60], the better the sound absorption values, especially in the range of low frequencies [61]. Generally, the acoustic properties of ASR-based materials can be improved in terms of sample thickness, which will be evaluated in detail and verified in further studies.

Due to relatively low sound absorption coefficients measured for all AR-based materials, the additional parameter, which enables assessment of the sound absorption ability for engineering purposes—mainly average sound absorption coefficient (α_avg_)—was calculated [16]. Based on the values of the absorption coefficients in the octave bands, the α_avg_ value was determined according to the formula

(2) $$\alpha_{avg}=\frac{1}{n}\overset{n}{\underset{i=1}{\sum }}\alpha_{f\left(i\right)}$$

where: *α*_*f*(*i*)_ is the sound absorption coefficients measured at the center frequencies *f*(*i*) (e.g., 100 Hz … 4000 Hz), and *n* is the number of octave bands taken into account.

**Figure 12:**
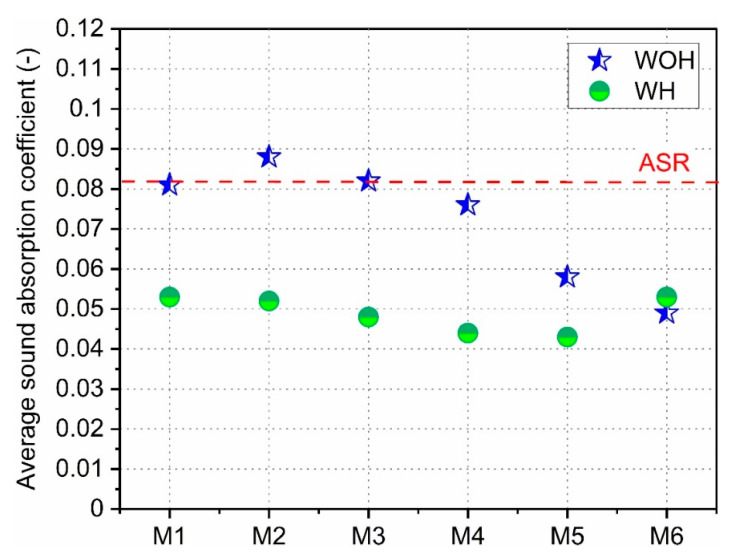
Average sound absorption coefficients of ASR-based materials manufactured with different compatibilization.

In Figure 12, the average sound absorption coefficient values calculated according to Equation (2) for all considered material series are presented. 

As a red dashed line, the reference sample made of non-compatibilized ASR is presented. In the case of samples homogenized (WH) using the rotary blades, it is clear that all material series had comparable acoustic properties. A clear downward trend can be observed in the case of non-homogenized samples (WOH). While the addition of maleic anhydride did not change the acoustic properties, the increasing proportion of polyethylene (series M4–M6 WOH) caused a gradual decrease in α_avg_. This is likely due to the increased proportion of low melting void-filling polymer in compressed ASR-based compositions.

## 4. Conclusions

The paper presents the new solution for polymer waste recycling, mainly recycling cables from end-of-life vehicles in Poland. Moreover, it was shown that the ASR-based compositions with various compatibilization procedures—including physical, chemical, and shear-assisted compatibilization—were successfully applied and verified by evaluating final product properties. The mechanical results obtained demonstrate the beneficial effect of shear-assisted homogenization, resulting in improved values of the tensile modulus and strength compared to composites without homogenization. The tensile modulus highest value was noted for the M5 WH series, containing 20 wt % rLDPE and 5 wt % maleic anhydrite-based compatibilizers. The most crucial effect on mechanical performance is physical compatibilization, mainly incorporation of additional ductile phase (rLDPE) in the amount above 20 wt %, both in the case of samples manufactured with additional high shear melt processing procedure. Changes are also noticeable in the structure of the tested materials. Samples with homogenization (WH) show a small amount of free space compared to samples without homogenization due to good adhesion between the composite components. The optimal solution for management of cable waste is the manufacture of ASR composites with homogenization using an internal mixer adding 20 wt % of rLDPE with the content of 5 wt % of a compatibilizer to the mixed plastics from cable waste.

Concluding, the results of this research have shown that applying a complex compatibilization method—consisting of the application of chemical, physical, and thermomechanical procedures—gives the most beneficial results. The simultaneous use of chemical compatibility with the introduction of an additional ductile phase to the ASR matrix in the form of post-consumer rLDPE waste allowed for obtaining products with favorable mechanical and thermomechanical properties, and low water absorption. Due to the obtained properties, a new and cheap composite material, especially M4–M6_WH with favorable mechanical and functional properties—i.e., increased strength and low water absorption—making it easy to clean for the production of anti-slip panels or wall claddings for the safety of animals in stables and cowsheds. The results showed that, thanks to the developed technology, it is possible to obtain ASR composites without separation based on mixed wastes with favorable mechanical properties.

The results obtained demonstrate that the hot-pressing with the pre-blending with rLDPE and compatibilizer of the ASR-based waste provides a high gain in mechanical and usage properties, enabling the circular economy of plastics from automotive cables.

## Figures and Tables

**Table 1:** Sample symbols and the percentage amount of ASR composite components.

Abbreviation	Sample	Content ASR wt %/K/LDPE
M0	ASR	100/0/0
M1	ASR +1K	99/1/0
M2	ASR + 2K	98/2/0
M3	ASR + 5K	95/5/0
M4	ASR + 5K + 10	85/5/10
M5	ASR + 5K + 20	75/5/20
M6	ASR + 5K + 50	45/5/50

## Data Availability

The data presented in this study are available on request from the corresponding author.

## Supplementary Materials

The following are available online at https://www.mdpi.com/article/10.3390/polym13213845/s1, Figures S1–S13: Selected FTIR-ATR spectra taken from ASR and M1–M6 samples, Figure S14: 100 mm samples used for acoustic properties determination, Table S1: FTIR-ATR spectra identification matches for selected points of compression-molded samples (average percentage value from 30 measurements).
