# Supplementary material for: Recycling of Plastics from Cable Waste from Automotive Industry in Poland as an Approach to the Circular Economy
Source: Polymers (Basel). 2021 Nov 7;13(21):3845. doi: 10.3390/polym13213845 (PMC8588454; doi:10.3390/polym13213845)
Supplement: Supplementary file 1 [file polymers-13-03845-s001.zip › polymers-1428218-supplementary.pdf]

Supplementary information

Recycling of Plastics from Cable Waste from Automotive In-dustry in Poland as an Approach to the Circular Economy

Dorota Czarnecka-Komorowska [1,*], Wiktoria Kanciak [1], Mateusz Barczewski [1], Roman Barczewski [2], Roman Regulski [3], Dariusz Sędziak [3] and Cezary Jędryczka [4]

[1] Faculty of Mechanical Engineering, Poznan University of Technology, 60-965 Poznan, Poland; wiktoria.kanciak@student.put.poznan.pl (W.K.), mateusz.barczewski@put.poznan.pl (M.B.)
[2] Institute of Applied Mechanics, Poznan University of Technology, 60-965 Poznan, Poland; roman.barczewski@put.poznan.pl
[3] Institute of Mechanical Technology, Poznan University of Technology, 60-965 Poznan, Poland; roman.regulski@put.poznan.pl (R.R.); dariusz.sedziak@put.poznan.pl (D.S.)
[4] Institute of Electrical Engineering and Electronics, Poznan University of Technology, 60-965 Poznan, Poland; cezary.jedryczka@put.poznan.pl
* Correspondence: dorota.czarnecka-komorowska@put.poznan.pl; Tel.: +48-(61)-665-2732

Supplementary information

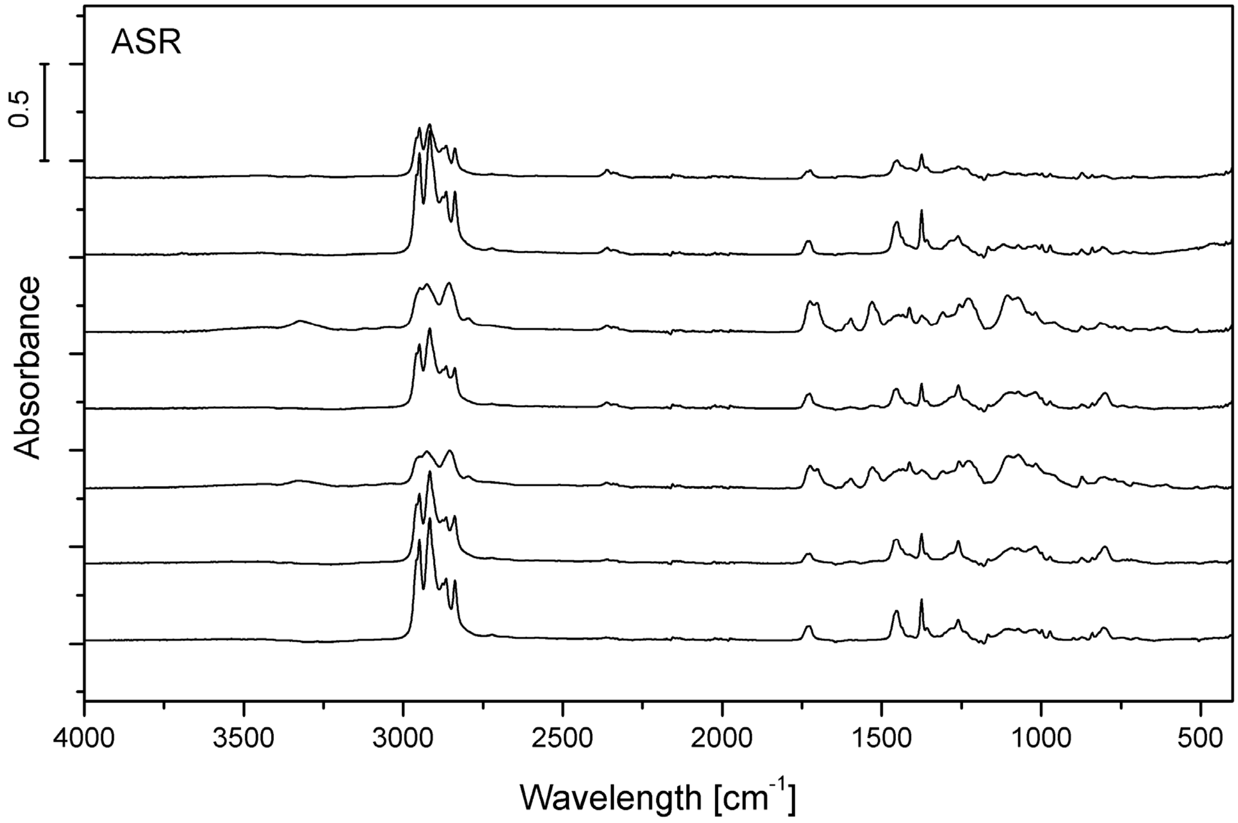

Figure S1. Selected FTIR-ATR spectra taken from ASR compression molded samples.

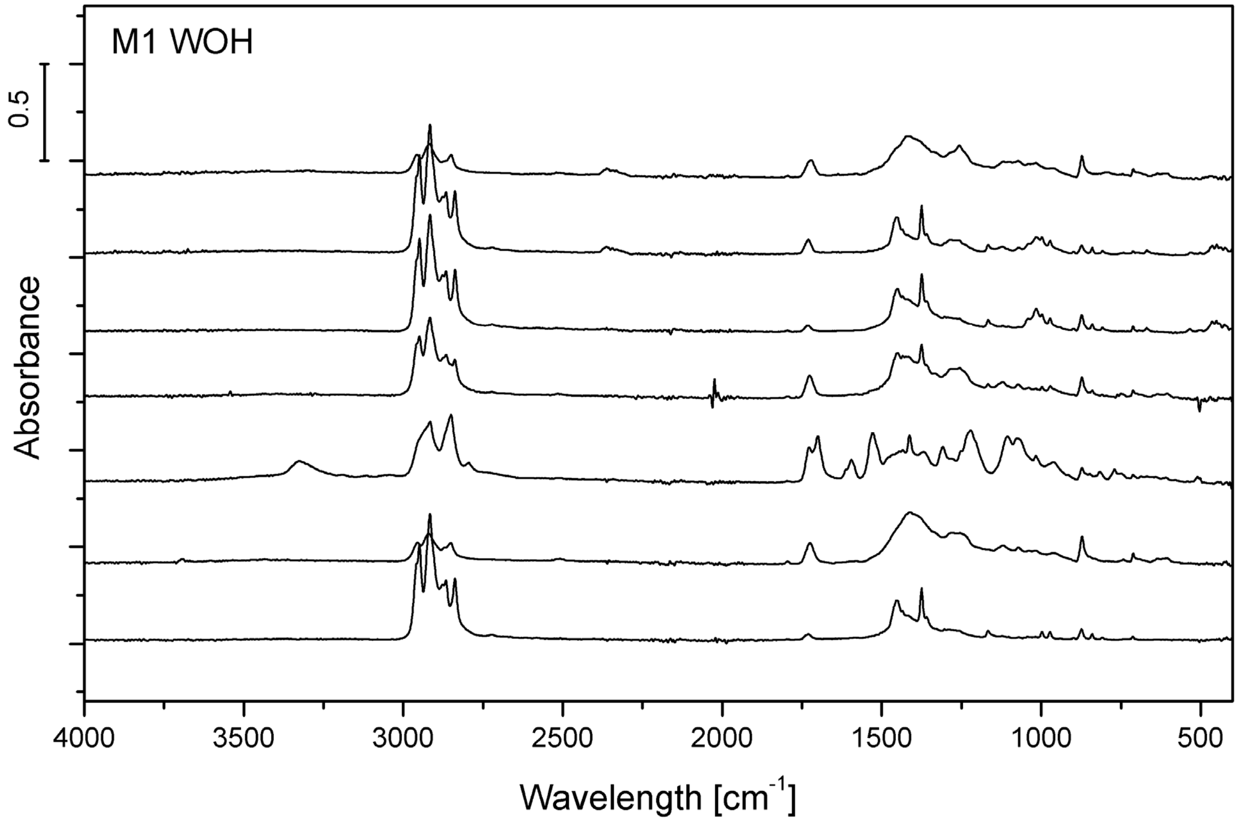

Figure S2. Selected FTIR-ATR spectra taken from M1 WOH compression molded samples.

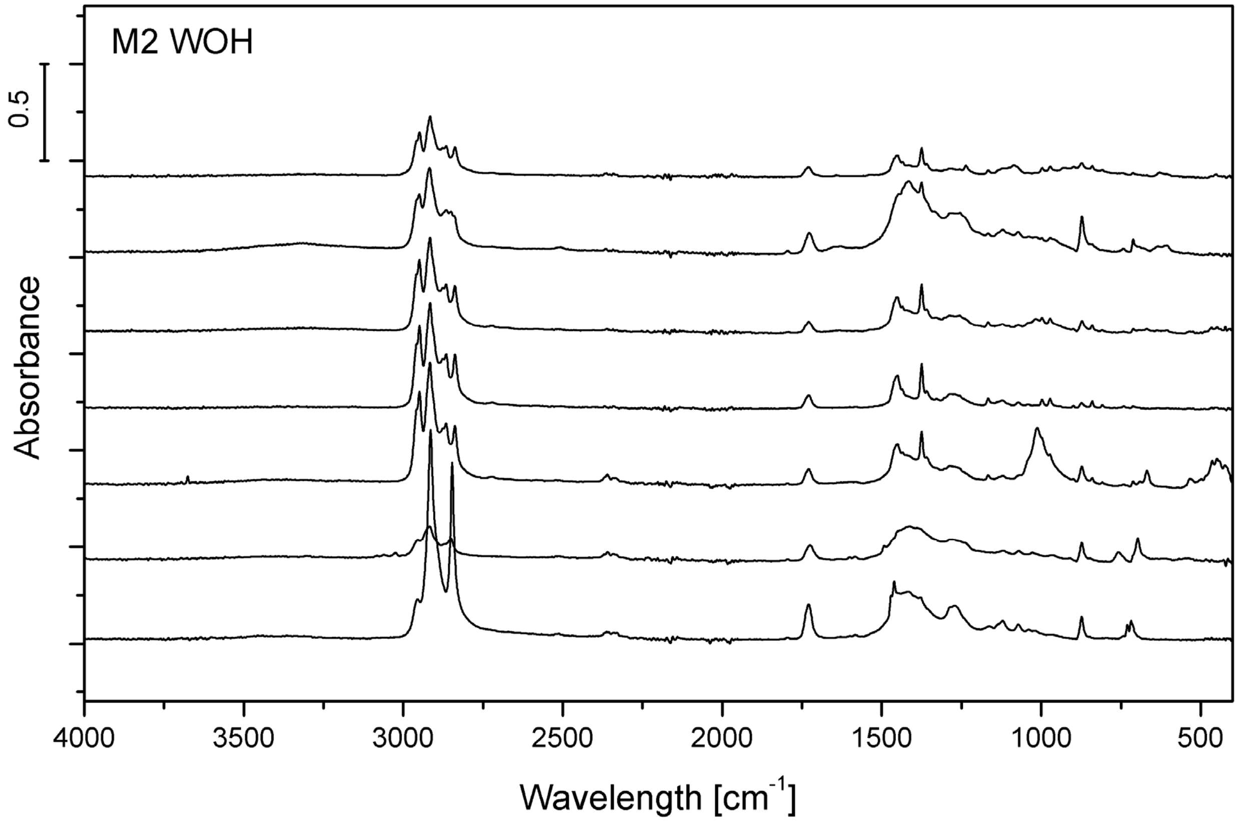

Figure S3. Selected FTIR-ATR spectra taken from M2 WOH compression molded samples.

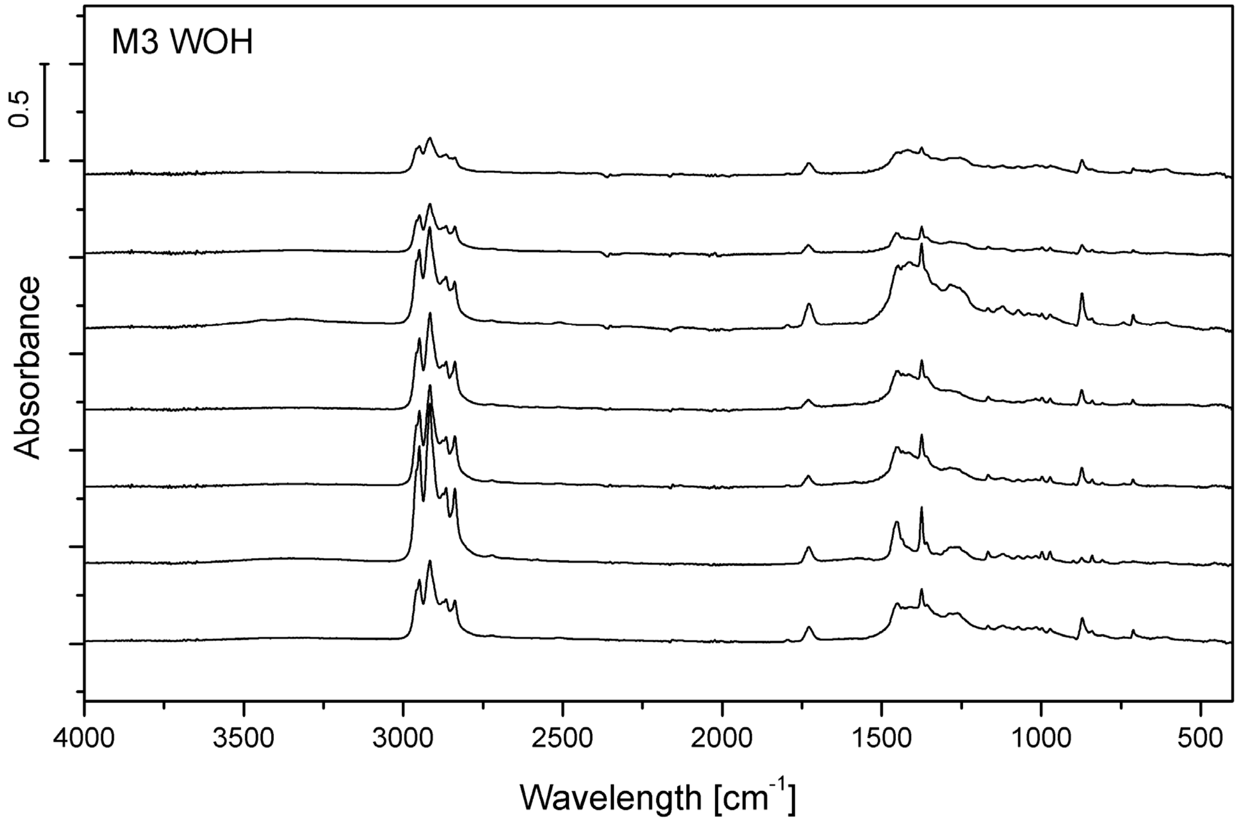

Figure S4. Selected FTIR-ATR spectra taken from M3 WOH compression molded samples.

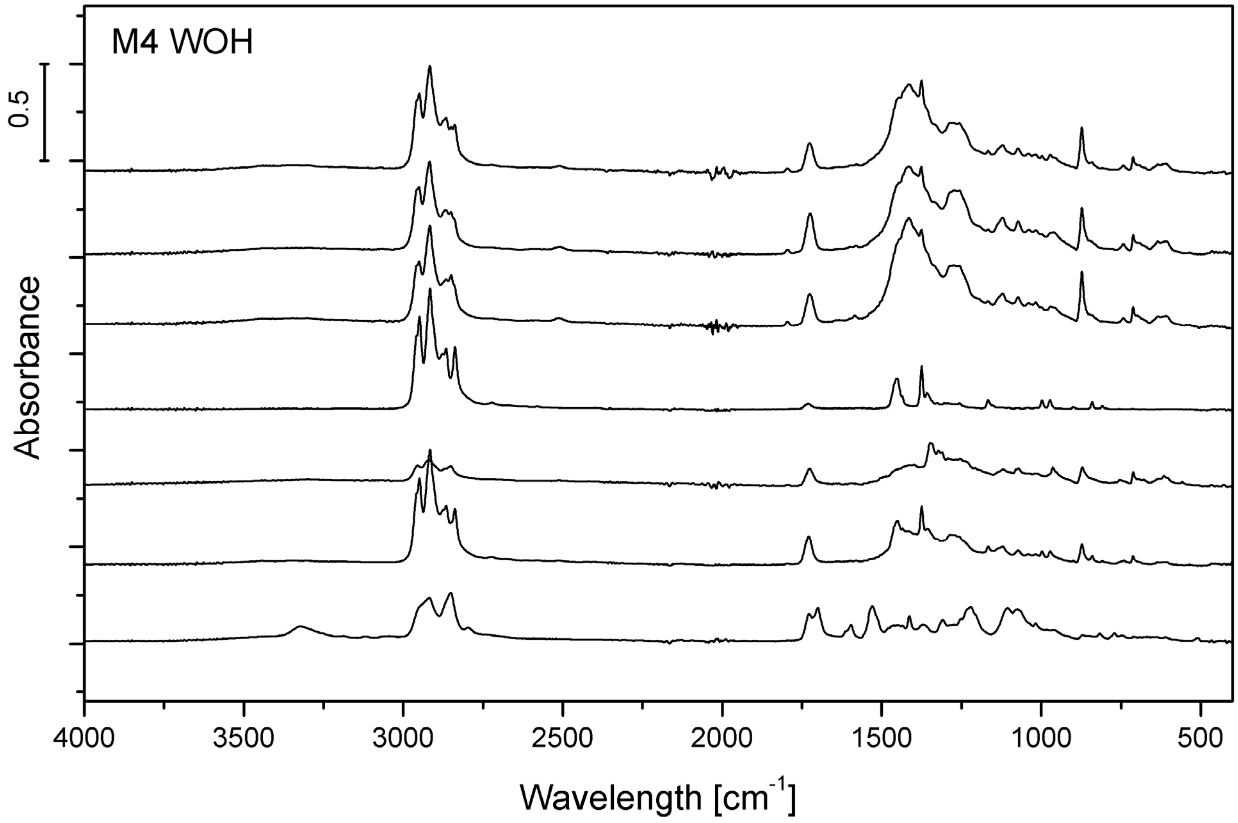

Figure S5. Selected FTIR-ATR spectra taken from M4 WOH compression molded samples.

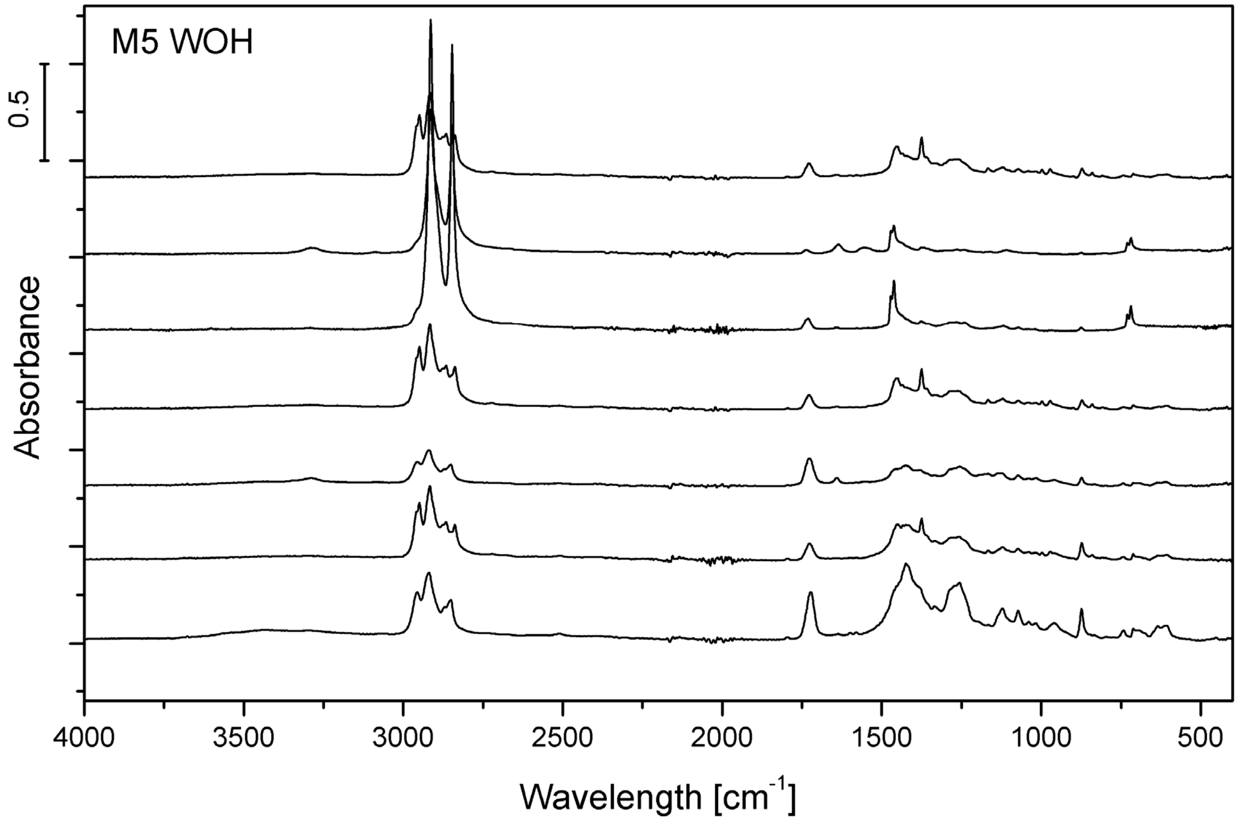

Figure S6. Selected FTIR-ATR spectra taken from M5 WOH compression molded samples.

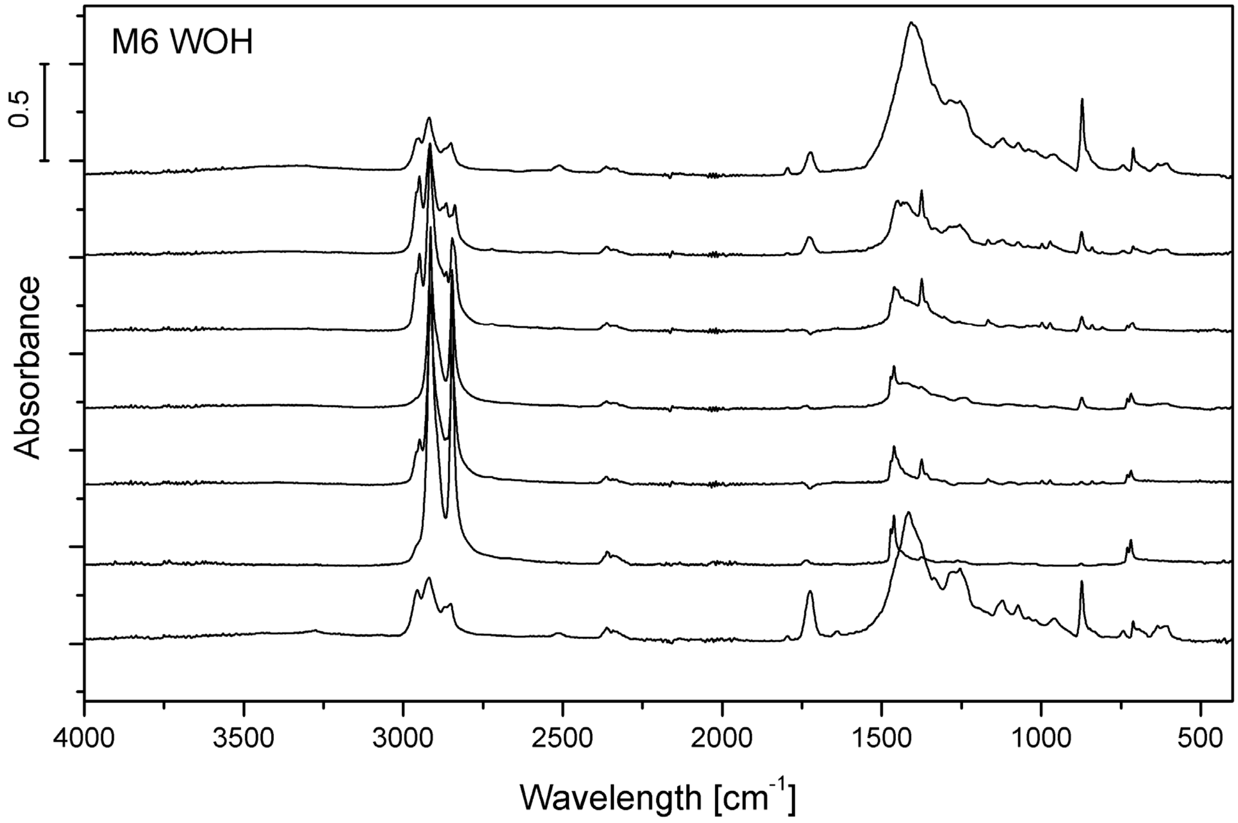

Figure S7. Selected FTIR-ATR spectra taken from M6 WOH compression molded samples.

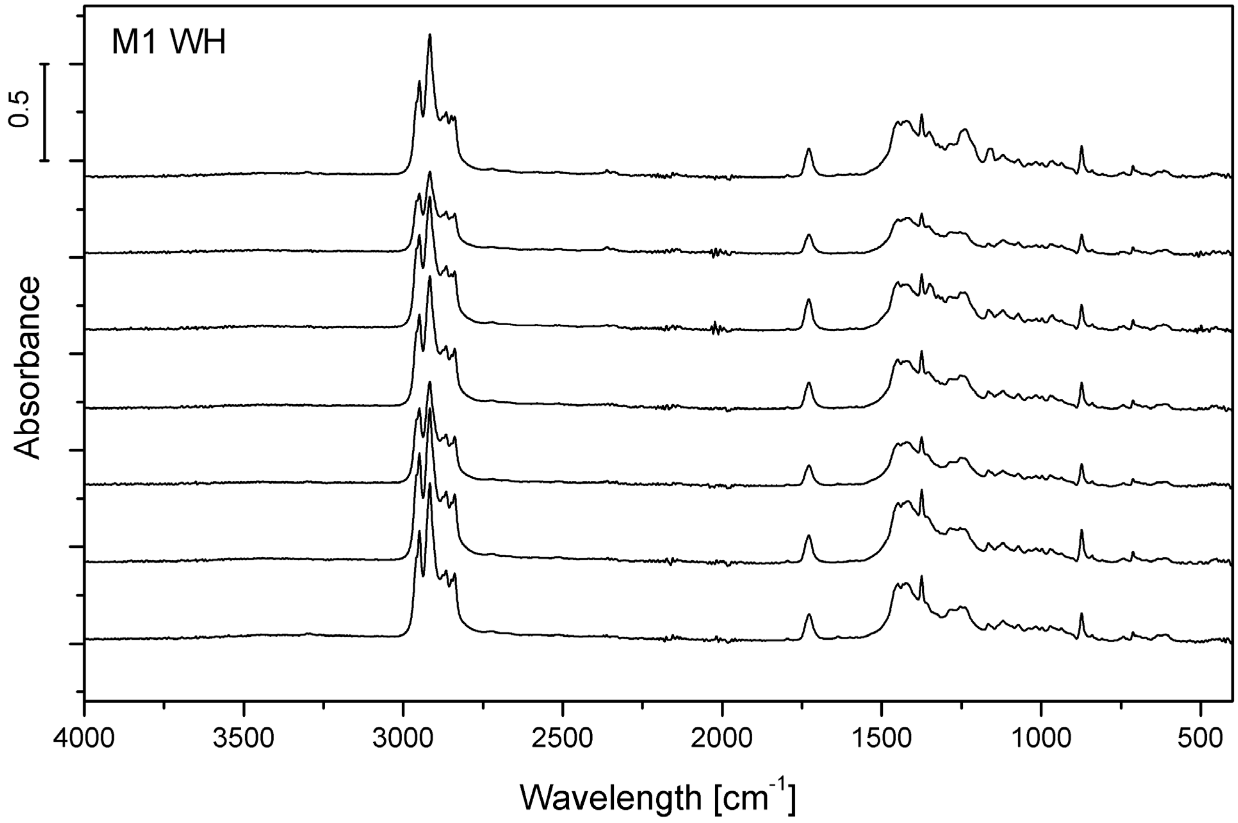

Figure S8. Selected FTIR-ATR spectra taken from M1 WH compression molded samples.

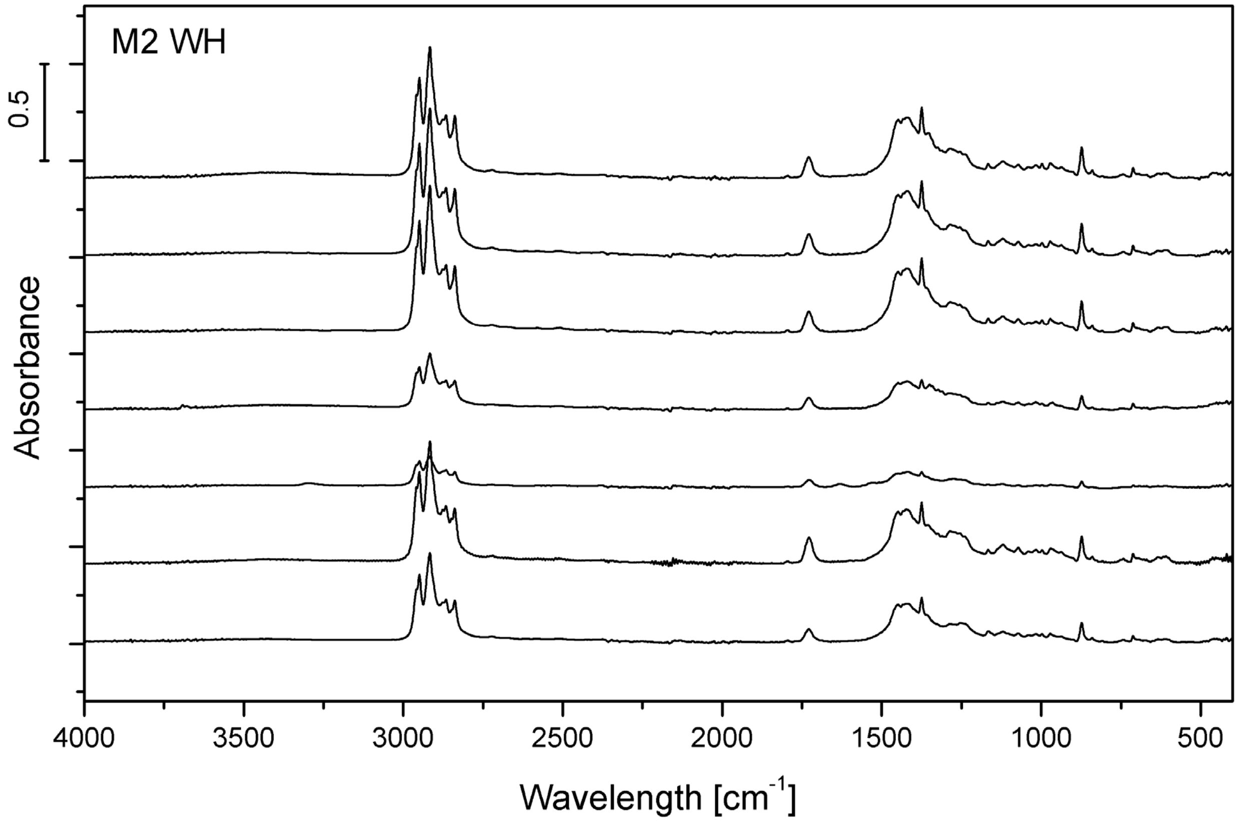

Figure S9. Selected FTIR-ATR spectra taken from M2 WH compression molded samples.

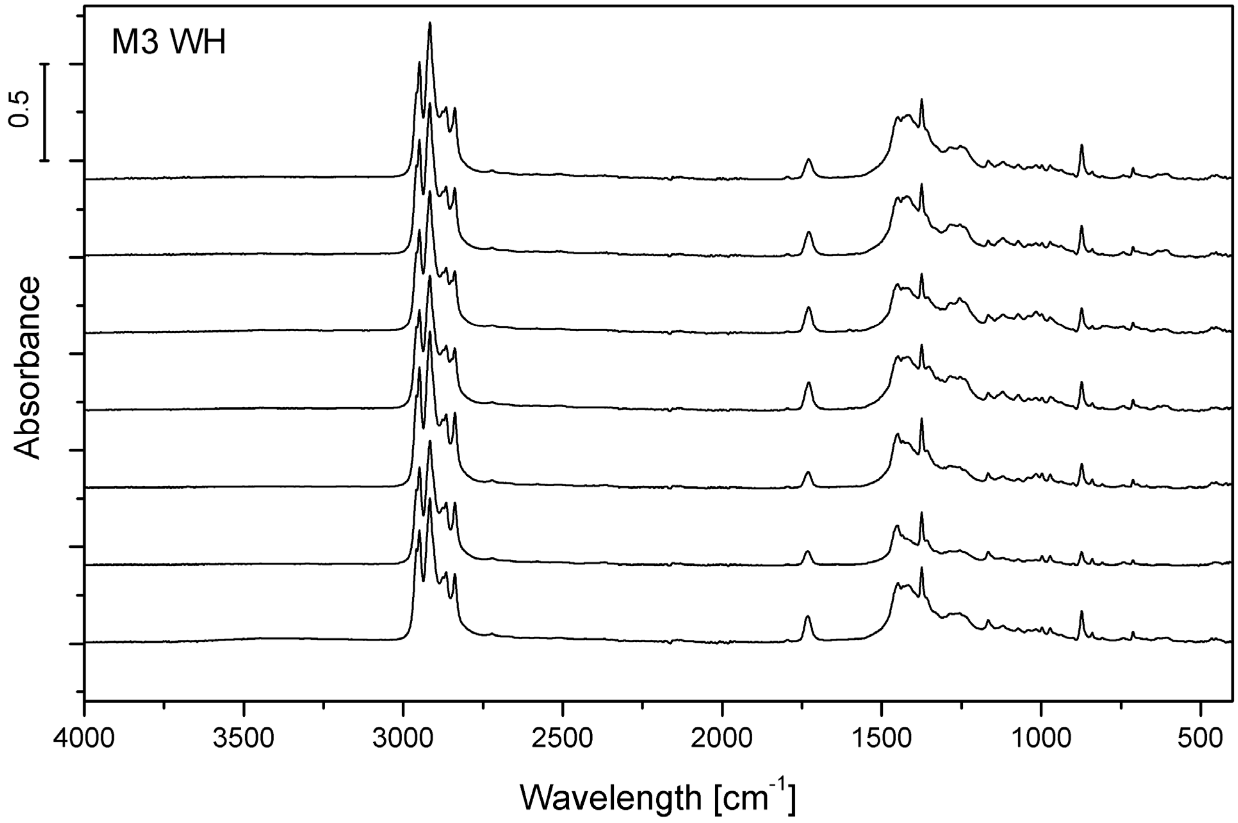

Figure S10. Selected FTIR-ATR spectra taken from M3 WH compression molded samples.

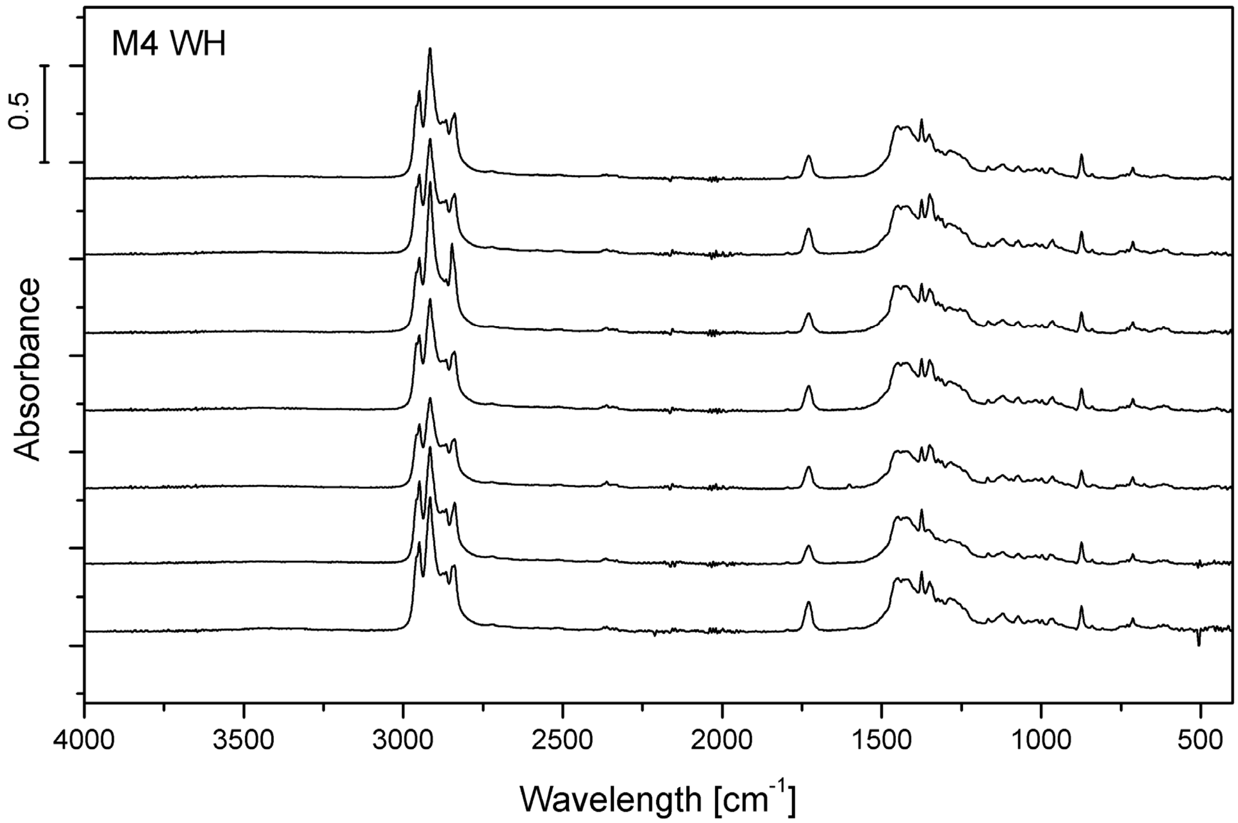

Figure S11. Selected FTIR-ATR spectra taken from M4 WH compression molded samples.

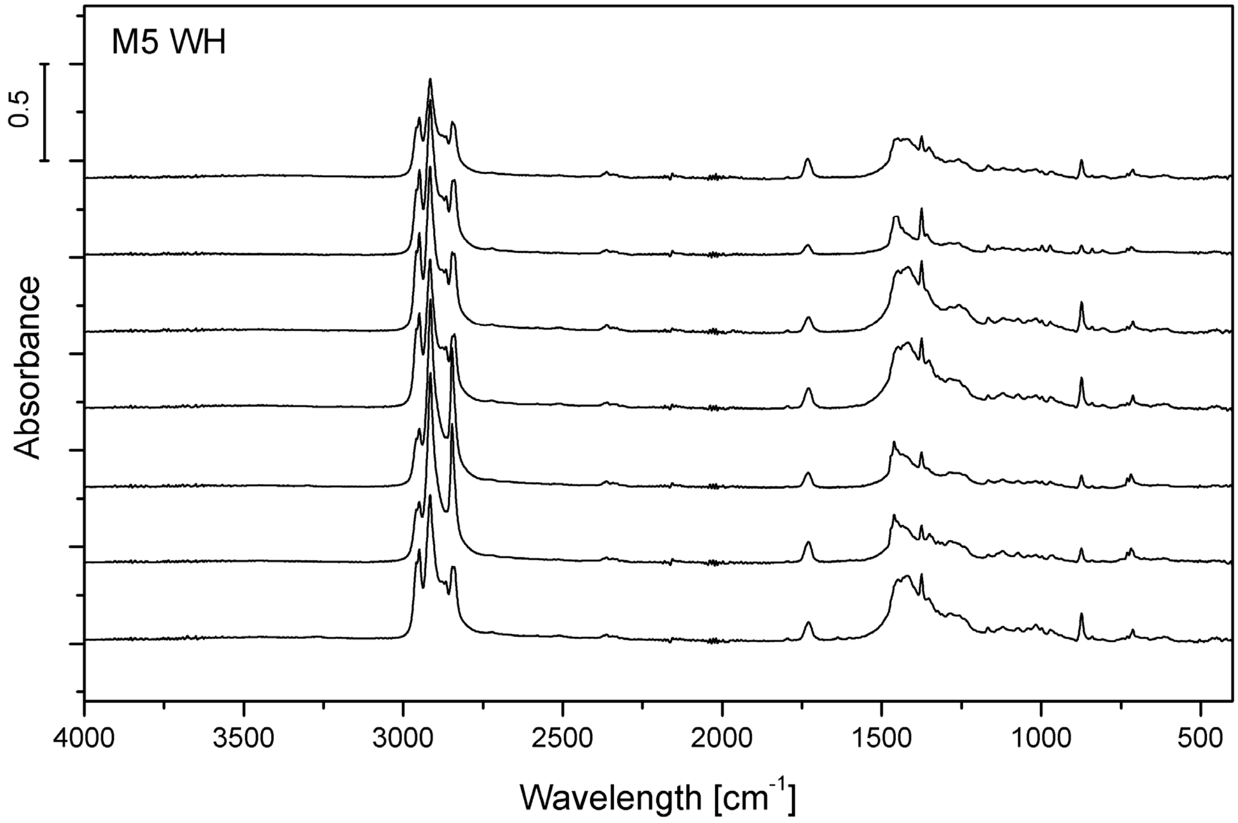

Figure S12. Selected FTIR-ATR spectra taken from M5 WH compression molded samples.

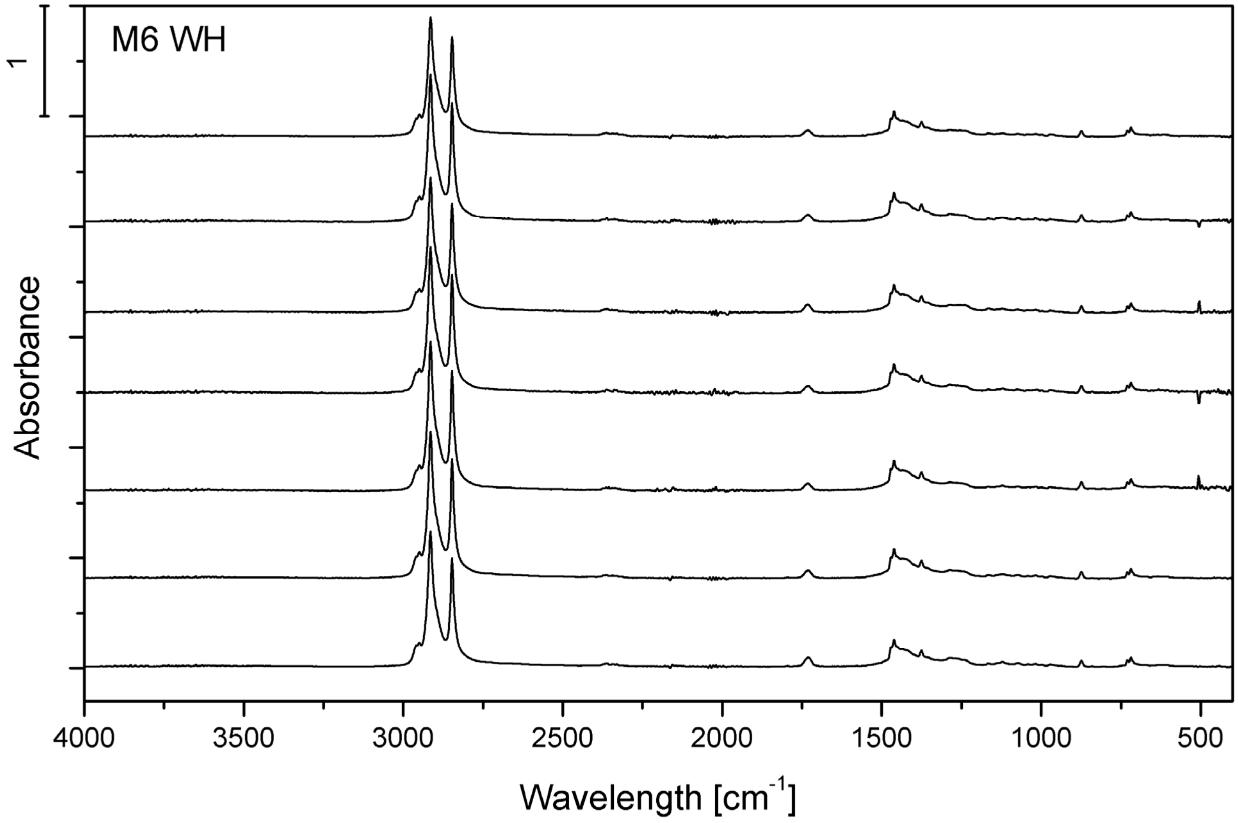

Figure S13. Selected FTIR-ATR spectra taken from M6 WH compression molded samples.

Table S1. FTIR-ATR spectra identification matches for selected points of compression-molded samples (average percentage value from 30 measurements).

Sample	Match 1	Match 2	Residual matches
ASR	poly(vinyl chloride) (47%)	cis-1,4-polyisoprene rubber (21%)	polybutene<br>polyurethane
M1 WOH	poly(vinyl chloride) (42%)	cis-1,4-polyisoprene rubber (16%)	polypropylene<br>polybutene<br>hydrogenated styrene/butadiene<br>polyurethane
M2 WOH	poly(vinyl chloride) (54%)	cis-1,4-polyisoprene rubber (12%)	high-density polyethylene<br>polybutene<br>hydrogenated styrene/butadiene<br>polypropylene
M3 WOH	poly(vinyl chloride) (42%)	cis-1,4-polyisoprene rubber (16%)	polybutene<br>polyether block copolymer<br>vinyl resin<br>polyurethane<br>polyester<br>trans-1,4-polyisoprene
M4 WOH	poly(vinyl chloride) (63%)	polyurethane (8%)	polyester<br>cis-1,4-polyisoprene rubber<br>trans-1,4-polyisoprene<br>polyacrylonitrile
M5 WOH	poly(vinyl chloride) (66%)	polyester block copolymer (11%)	polyether imide<br>vinyl resin
M6 WOH	poly(vinyl chloride) (33%)	cis-1,4-polyisoprene rubber (33%)	high-density polyethylene<br>trans-1,4-polyisoprene<br>polyetherimide<br>vinyl resin
M1 WH	poly(vinyl chloride) (35%)	cis-1,4-polyisoprene rubber (30%)	polybutene<br>hydrogenated styrene/butadiene
M2 WH	cis-1,4-polyisoprene rubber (33%)	poly(vinyl chloride) (25%)	polybutene<br>polyethylene terephthalate<br>hydrogenated styrene/butadiene<br>trans-1,4-polyisoprene
M3 WH	cis-1,4-polyisoprene rubber (34%)	poly(vinyl chloride) (23%)	hydrogenated styrene/butadiene<br>trans-1,4-polyisoprene<br>polybutene<br>polypropylene
M4 WH	cis-1,4-polyisoprene rubber (33%)	hydrogenated styrene/butadiene (33%)	poly(vinyl chloride)<br>polybutene
M5 WH	cis-1,4-polyisoprene rubber (33%)	hydrogenated styrene/butadiene (24%)	high density polyethylene<br>polybutylene<br>trans-1,4-polyisoprene
M6 WH	high density polyethylene (58%)	cis-1,4-polyisoprene rubber (31%)	polybutene<br>trans-1,4-polyisoprene

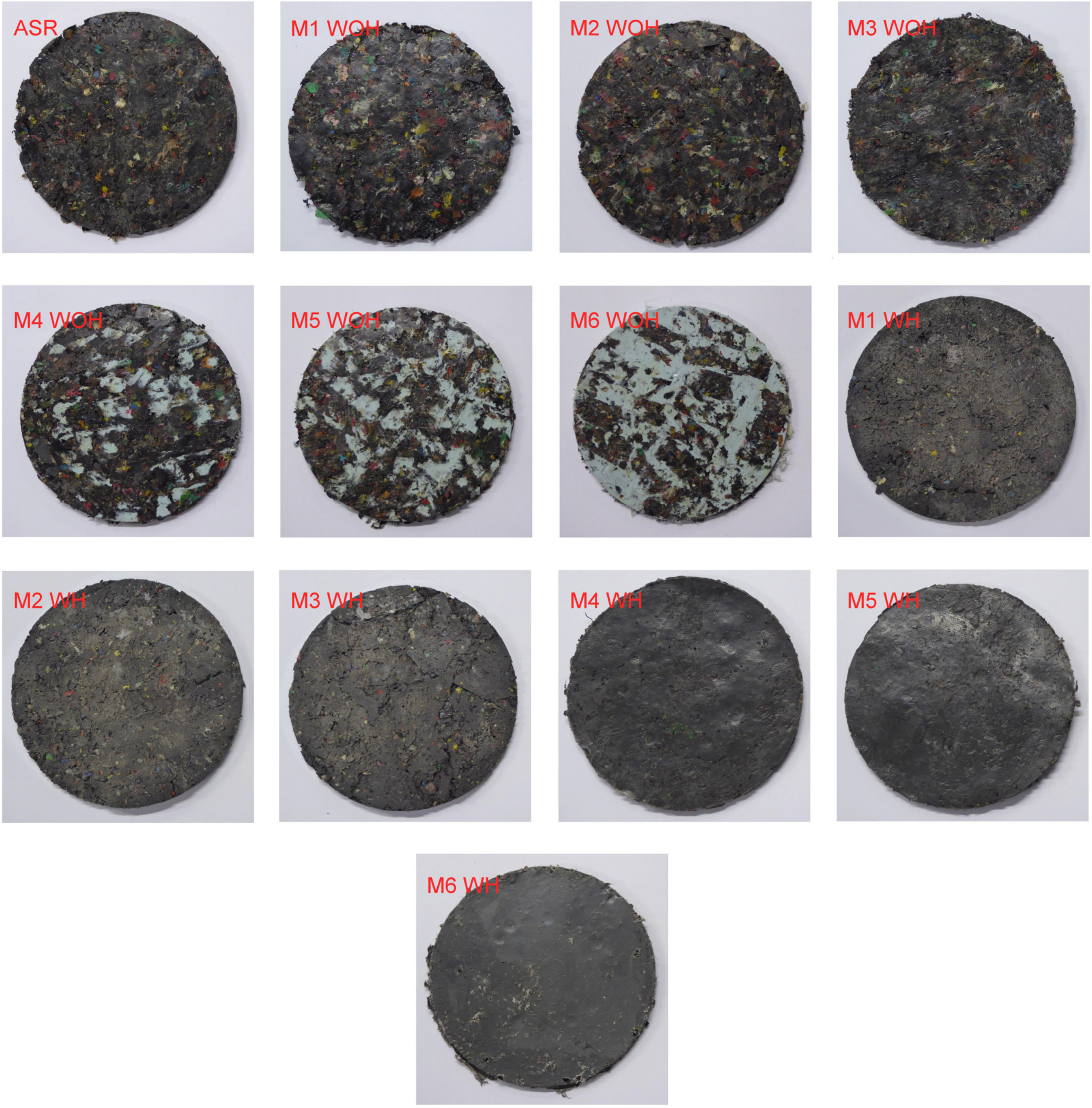

Figure S14. 100 mm samples used for acoustic properties determination.
